# Giant gastric stromal tumor mimicking as a posterior mediastinal mass: A case report and literature review

**DOI:** 10.1097/MD.0000000000012816

**Published:** 2018-10-12

**Authors:** Xiaonan Yin, Chaoyong Shen, Yuan Yin, Zhaolun Cai, Zhixin Chen, Bo Zhang

**Affiliations:** Department of Gastrointestinal Surgery, West China Hospital, Sichuan University, Chengdu, Sichuan, China.

**Keywords:** gastrointestinal stromal tumor, KIT, mediastinum

## Abstract

**Rationale:**

Gastrointestinal stromal tumors (GISTs) are the most common mesenchymal tumors of the gastrointestinal tract. Mediastinal GISTs are rare and mostly arise from the esophagus.

**Patient concerns:**

A 68-year-old woman with dysphagia who presented with a huge posterior mediastinal mass.

**Diagnoses:**

The patient was diagnosed with a GIST through chest computed tomography (CT)-guided core biopsy of the mass.

**Interventions:**

Complete excision including the tumor, lower part of the esophagus and fundus of the stomach were performed.

**Outcomes:**

On follow-up after 48 months, the patient is currently alive without any evidence of tumor recurrence.

**Lessons:**

The case highlights GISTs are taken into consideration in the differential diagnosis of posterior mediastinal masses.

## 1 Introduction

Gastrointestinal stromal tumors (GISTs) are the most common mesenchymal tumors of the gastrointestinal tract and are characterized by the gain-of-function mutations of KIT and platelet-derived growth factor alpha (PDGFRA) gene. GISTs most often arise in the stomach (60%–70%) and small intestine (25%–30%), but less frequently occur in colorectum (5%–15%), duodenum (5%), and esophagus (< 2%). Extraintestinal tumor locations such as omentum, mesentery, gallbladder, pancreas, and retroperitoneum are very rare.^[1–3]^ A small number of cases of mediastinal GISTs have been reported, and mostly originate from the esophagus.^[4–15]^ Only 2 cases of gastric GISTs in the posterior mediastinum have been reported.^[8,10]^ Here, we reported a rare case of a huge gastric GIST in a patient with dysphagia who presented with a posterior mediastinal mass. The patient was diagnosed as GISTs using core biopsy before operation, and subsequently was treated with radical resection.

## 2 Case report

**Figure 1:**
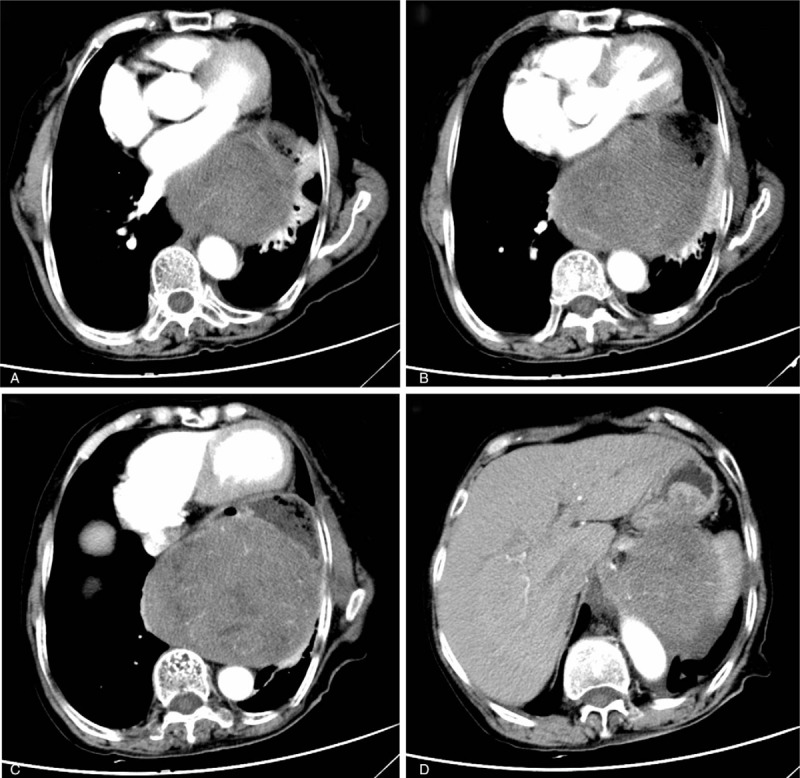
Contrast-enhanced abdominal computer tomography (CT) showed a heterogeneous, 11.9 × 10.2 cm posterior mediastinal tumor in the left lower thorax, thrusting the distal esophagus, pylorus, cardiac forward, pressing the pulmonary leading to the atelectasis of part of the upper and inferior lobe of left lung, and adhering to the lower esophagus, pylorus, and the left adrenal gland with unclear boundaries.

A 68-year-old woman presented with progressively worsening dysphagia that occurred 5 months ago, with a rapid weight loss of 3 kg. She was previously healthy with no special medical history. Esophagogastroscopy showed a prominent luminal stenosis of the esophagus, which is apparently caused by an external pressure. A contrast-enhanced abdominal computed tomography (CT) revealed an inhomogeneous, soft tissue mass in the posterior mediastinum measuring 11.9 × 10.2 cm (Fig. 1). Tumor markers including CEA, CA19-9, CA125, were all within the normal range.

The patient underwent a mediastinoscopy and chest CT-guided core biopsy of the mass. Biopsy was suspicious of stromal tumor. Immunohistochemical stains of tumor cells were consistent with a GIST (strong positive for CD117, CD34, and DOG-1; negative for SMA, PCK, CK18, S-100, and desmin; Ki67 index was 8%). Molecular analysis revealed a deletion mutation in exon 11 of the KIT gene.

The patient was counseled receiving preoperative imatinib therapy and the second-stage operation. She refused and insisted direct surgical resection. During the operation, we found that the mass pressed the inferior lobe of the left lung, distal esophagus, thoracic aorta and was adherent to the fundus of stomach, which was in favor of gastric origin instead of the esophagus. Curative resection including the tumor, lower part of the esophagus, and fundus of the stomach were performed. The tumor measured 13 × 10 × 10 cm and was well-encapsulated, firm mass with focal hemorrhage and cystic formation, and without obvious necrosis. The microscopic findings showed that the tumor cells were spindle in shape; the mitotic counts were increased to 18 per 50 high-power fields. Immunohistochemical analysis of tumor cells confirmed a high-risk GIST according to the National Institutes of Health (NIH) risk criteria, which was consistent with the previous report. The postoperative course was uneventful, without adjuvant imatinib therapy. Forty-eight months postoperative, the patient is alive without any evidence of tumor recurrence.

## 3 Discussion

GISTs are rare tumors that occur in approximately 0.1% to 3.0% of patients with gastrointestinal neoplasms. Historically, GISTs had always been diagnosed as leiomyoma, leiomyosarcoma, leiomyoblastoma, and schwannoma until the concept of gastric stromal tumor was introduced by Mazur and Clark in 1983.^[16]^ GISTs arise from Cajal cells of the gastrointestinal tract or their precursors. The morphology of GISTs shows that a majority of GISTs consist of uniform population of spindled cells, 20% of the tumors have an epithelial morphology, and 10% are a mixture of spindle and epithelial cells. The combination of CD117 and DOG1 has helped define the diagnosis of most GISTs.^[17]^

The common differential diagnoses in the posterior mediastinum include neurogenic tumors, bronchogenic cysts, smooth muscle tumors, and type A thymoma. In this case, CT showed a heterogeneous, 11.9 × 10.2 cm mass in the posterior mediastinum. The mass infiltrated generally of adjacent structures and manifested the features of malignant tumors. To make a definitive diagnosis, preoperative core biopsy was carried out. The results of immunohistochemistry confirmed a gastric GIST. Mediastinal GISTs are very rare, most of which originate from the esophagus. A comprehensive review of 12 case reports (Table 1) and case series with mediastinal GISTs showed that most arose from the esophagus and only 2 cases originated from the stomach, mediastinal GISTs mostly occurred in the elderly and manifested as giant masses, the clinical presentations were complex and varied. In all the 12 cases, R0 resection was performed. However, only 1 case reported by Constantinoiu et al^[4]^ was given adjuvant imatinib after surgery. Follow-up data was available in 9 cases (9/12, 75%). Median follow-up was 24 months, and all patients were disease free.

**Table 1:**
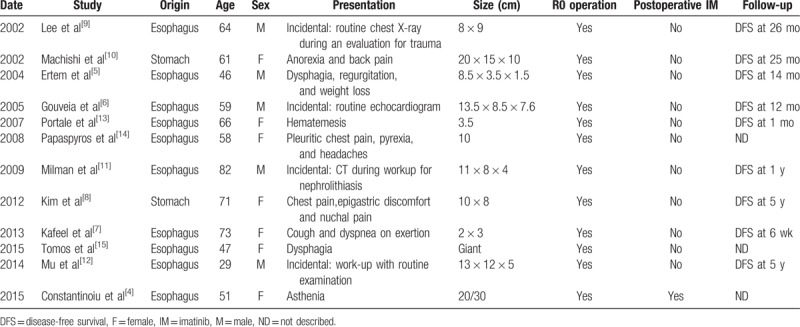
Summary of GISTs presented with posterior mediastinal masses.

The vast majority of GISTs harbor gain-of-function mutations of KIT (75%–80%). Mutations in KIT exon 11 are the most common mutations found in GIST and arise mostly from the stomach, followed by KIT exon 9 mutations arising most frequently in the small and large intestine. The application of a small-molecular tyrosine kinase inhibitor (TKI), imatinib, has revolutionized the treatment of GISTs, resulting in prolonged recurrence-free survival (RFS) and overall survival (OS) in patients with advanced, inoperable, or high-risk disease.^[18]^ KIT exon 11 mutations in GIST have the highest responses to imatinib at an effective dose of 400 mg/d, whereas KIT exon 9 mutations need 800 mg/d imatinib to achieve a clinical response. It has been reported that preoperative imatinib can shrink the size of tumor, prevent tumor rupture, and facilitate complete and less invasive resection during surgery.^[19]^ This may result in an improved survival in patients with locally advanced primary GIST. In this case, the tumor infiltrated generally into adjacent structures and R0 resection was difficult to achieve. Molecular analysis revealed that the tumor harbored a deletion mutation in exon 11 of KIT, and preoperative imatinib therapy was recommended. However, the patient refused and an extensive resection including the tumor, lower part of the esophagus, and fundus of the stomach was performed.

Adjuvant imatinib therapy following surgery is necessary for patients with intermediate and high-risk GIST. ACOSOG Z9001 trial^[20]^ revealed that patients with 1-year adjuvant imatinib had less recurrent events compared with those receiving placebo. SSGXVIII/AIO trial^[21]^ demonstrated that 3-year adjuvant imatinib in high-risk patients prolonged RFS and OS compared with 1-year imatinib treatment. However, optimal duration of adjuvant imatinib in GIST patients with complete resection remains controversial. Lin et al^[22]^ suggested that patients with high-risk GISTs receive imatinib treatment for at least 5 years. Unfortunately, the patient in this case did not receive imatinib treatment.

In conclusion, we reported a rare case of gastric GIST in a patient who presented with a huge posterior mediastinal mass. Our study highlights that GISTs need to be considered in the differential diagnosis of posterior mediastinal masses. For initially unresectable mass that is suspicious of GIST, a preoperative biopsy and molecular gene test are recommended.

## Acknowledgments

The authors gratefully acknowledge the whole staff of the Department of Gastrointestinal Surgery, West China Hospital, who generously provided assistance in the collection of data throughout the duration of the study.

## Author contributions

**Conceptualization:** Chaoyong Shen, Bo Zhang.

**Data curation:** Xiaonan Yin.

**Formal analysis:** Xiaonan Yin.

**Funding acquisition:** Bo Zhang.

**Investigation:** Chaoyong Shen, Yuan Yin.

**Methodology:** Chaoyong Shen, Zhaolun Cai.

**Project administration:** Yuan Yin.

**Resources:** Yuan Yin.

**Software:** Zhaolun Cai.

**Supervision:** Yuan Yin.

**Validation:** Zhixin Chen, Bo Zhang.

**Visualization:** Chaoyong Shen, Zhaolun Cai, Zhixin Chen.

**Writing – original draft:** Xiaonan Yin.

**Writing – review & editing:** Zhixin Chen, Bo Zhang.

Bo Zhang orcid: 0000-0002-0254-5843.
